# Global Analysis of CPEBs Reveals Sequential and Non-Redundant Functions in Mitotic Cell Cycle

**DOI:** 10.1371/journal.pone.0138794

**Published:** 2015-09-23

**Authors:** Valeria Giangarrà, Ana Igea, Chiara Lara Castellazzi, Felice-Alessio Bava, Raul Mendez

**Affiliations:** 1 Institute for Research in Biomedicine (IRB Barcelona), Barcelona, Spain; 2 Institució Catalana de Recerca i Estudis Avançats (ICREA), Barcelona, Spain; University of British Columbia, CANADA

## Abstract

CPEB (Cytoplasmic Polyadenylation Element Binding) proteins are a family of four RNA-binding proteins that regulate the translation of maternal mRNAs controlling meiotic cell cycle progression. But CPEBs are not limited to the transcriptionally silent germline; they are also expressed, in various combinations, in somatic cells, yet their role in regulation of mitosis-related gene expression is largely unknown. Deregulation of CPEB1 and CPEB4 have been linked to tumor development. However, a systematic analysis addressing their requirements for the temporal regulation of mitotic gene expression has yet to be performed. This study addresses the requirements of each of the four CPEBs for mitotic phase transitions, with a particular focus on cytoplasmic polyadenylation and translational regulation. We demonstrate that CPEB3 is the only member dispensable for mitotic cell division, whereas the other three members, CPEB1, 2, and 4, are essential to successful mitotic cell division. Thus, CPEB1 is required for prophase entry, CPEB2 for metaphase and CPEB4 for cytokinesis. These three CPEBs have sequential non-redundant functions that promote the phase-specific polyadenylation and translational activation of CPE-regulated transcripts in the mitotic cell cycle.

## Introduction

Cytoplasmic changes in poly(A) tail length regulate the translation of mRNAs in many biological contexts [1–4]. Cytoplasmic poly(A) tail elongation is mediated primarily by a cis-acting element, named the Cytoplasmic Polyadenylation Element (CPE), present in the 3’-UTR of the regulated transcripts. This element is targeted by CPE-Binding Proteins (CPEBs), which have RNA-binding capabilities. CPEBs recognize overlapping mRNA populations, although with different affinities, but are differentially regulated through the divergent N-terminal regulatory domains [5–9]. This combination of a common RNA-binding domain with unique regulatory elements could define the phase-specific requirements for CPEBs. Thus, in meiotic *Xenopus Laevis* oocytes, sequential expression and phosphorylation of CPEB1 and CPEB4 sustain the temporal and spatial regulation of gene expression defined by the combinatorial code of CPEs [7, 10–13]. Although in somatic mitosis CPEBs function(s) have been studied in much less detail, phase-specific changes in poly(A) tail length have been reported [6]. Moreover, in tumor cells, depletion of CPEB1 disrupts this mitotic cytoplasmic regulation of poly(A) tail length, as well as pre-mRNA nuclear alternative polyadenylation site selection, and inhibits cell proliferation [6, 14]. On the contrary, in primary fibroblasts, CPEB1 depletion promotes senescence bypass [15]. CPEB4 depletion in tumor cells has limited impact on cell proliferation but prevents growth of xenografted tumors[6, 16]. No functions in cell division have yet been defined for CPEB2 and 3. Thus, although both CPEB1 and CPEB4 have been linked to cell proliferation and tumor development, the evidence is conflicting as to whether they act as tumor suppressors or oncogenes based on their role in cell cycle progression. In part, these apparent discrepancies likely result from fragmented data as the four CPEB family members have yet to be studied simultaneously and in the same biological context.

In this study, we developed an inducible reporter system to systematically deplete each member of the CPEB family of proteins, with the goal to dissect the isolated role of each member individually. In HEK-293 cells, we observed that CPEB1, CPEB2, and CPEB4, but not CPEB3, have distinct and sequential roles required for proper control of cell proliferation. We found that CPEB1 is required for prophase entry, CPEB2 for metaphase-to-anaphase transition, and CPEB4 for cytokinesis and proper chromosomal segregation. Furthermore, using a dual GFP/RFP reporter that allows live analysis of polyadenylation and translation of CPE-regulated transcripts in the context of the cell cycle, we demonstrate that CPEB1, CPEB2, and CPEB4 are required to sustain specific polyadenylation dynamics during the M-phase of cell cycle, where a burst of GFP translation is observed. Taken together, our results provide the first global view of the cytoplasmic function of the four members of the CPEB family during the somatic cell cycle, clarifying their coordinated contribution to cell cycle regulation.

## Materials and Methods

### Antibodies

Anti-CPEB1 antibody was from Proteintech (13274-1-AP) and anti-CPEB2 from Abcam (ab126273). Anti-CPEB4 (NM_030627) rabbit polyclonal antibody was raised against amino acids 1–302 [6]. Anti-α-tubulin was supplied by Sigma (T902-6).

### Oligonucleotides- For RT-qPCR

hCPEB1, 5’- CACCTCTGCCCTTCCTGTC -3’ (sense) and 5’-CCAGGTACAGGTGGCTTCAT -3’ (antisense); hCPEB2, 5’-GGCTGTATGGTGGAGTTTGT -3’ (sense) and 5’- GGCATTCATCACACATCTGG -3’ (antisense); hCPEB3, 5’-AAAGCCCTTCTCCAGCAA -3’ (sense) and 5’- TGCAAAATGATTCCTCCACA -3’ (antisense); hCPEB4, 5’- AGCTTGCGATGATAATGGAT -3’ (sense) and 5’- CCCCTGACATTCATCACACA -3’ (antisense); α-galactosidase (housekeeping gene), 5’- CAGAAATCCGACAGTACTGCAA -3’ (sense) and 5’- CATATCTGGGTCATTCCAACC -3’ (antisense), mCPEB1, 5’- CCTCCTCTGCCCTTTCTTTC -3’ (sense) and 5’- TCCAAGAAGGTCCCAAGATG -3’ (antisense); mCPEB2, 5’- TGGAGCAACCATCAGAACAG -3’ (sense) and 5’- CGAGTGAATTTCGGTGGTG -3’ (antisense); mCPEB3, 5’- CGTTTGTACGGTGGTGTTTG-3’ (sense) and 5’- CCGTTTGTCAATGTCGTTGT-3’ (antisense); mCPEB4, 5’- TTGTTTCCGATGGAAGATGG-3’ (sense) and 5’- TCAATATCAGGAGGCAATCCA -3’ (antisense); α-galactosidase (housekeeping gene), 5’- ACCAGCAGGTGACACAGATG 3’ (sense) and 5’- GAAACAGTAGCCCTGCTTGC -3’ (antisense), GFP, 5’- ACGTAAACGGCCACAAGTTC -3’ (sense) and 5’-AAGTCGTGCTGCTTCATGTG -3’ (antisense); RFP, 5’- CGGCTCCTTCATCTACAAGG -3’ (sense) and 5’- GGTGATGTCCAGCTTGGAGT -3’ (antisense).

For Poly(A) tail assay: SP2, 5’-P-GGTCACCTCTGATCTGGAAGCGAC-NH2-3’ (sense), ASP2T, 5’- GTCGCTTCCAGATCAGAGGTGACCTTTTT-3’ (antisense), GFP, 5’- TCGAATTCTGTTGGCACCATGT -3’ (sense), RFP, 5’- ACCCTGCAGCCTGTGCTTCT -3’ (sense).

### Plasmid constructions

The enhanced green fluorescent protein (EGFP) from pEGFP-C1 was substituted with a destabilized green fluorescent protein with a fluorescence half-life of 2h (d2EGFP) from pNFkB-d2EGFP (Clontech Laboratories, Inc.) by using AgI and BglII restriction enzymes. Cyclin B1–Pum 3’UTR[11] was cloned downstream of d2EGFP. The SV40 polyadenylation signal from the pT7-dEGFPN1 plasmid was mutated from AATAAA to AAGGAA by site-directed mutagenesis. CPEs were mutated from TTTTAAT to TTgggAT, from TTTTACT to TTggACT, and from TTTTAAT to TTGGAAT. Destabilized red fluorescent protein with a fluorescence half-life of 8–12h (DsRFP) from pDsRed-Express-DR (Clontech Laboratories, Inc.) was subcloned in pFLAG-CMV-2, to add a functional promoter, by using HindIII and NotI. Then, DsRFP was subcloned in pLHCX using HindIII and ClaI restriction enzymes. pmKate2-H2B DNA plasmid was from Evrogen.

### Cell cultures and DNA transfections

Exponentially growing HEK-293 cells were cultured in Dulbecco's Modified Eagle Medium (DMEM), 10% FBS (fetal bovine serum) and 1% penicillin/streptomycin at 37°C in an atmosphere of 5% CO_2_. Cells were co-transfected at 50% confluence in 10-cm diameter dishes using a modified calcium phosphate method [17] and 10 μg of plasmid GFP DNA (CPE+ or CPE-) and RFP DNA and, when indicated, with pmKate2-H2B DNA plasmid (Evrogen). The GFP and RFP vectors were stably selected with 500 ug/ml Hygromicin B (for RFP) and 500 ug/ml G418 (for GFP) disulfate salt solutions. Double-positive cells (GFP-RFP) were selected by cell sorting.

When needed, cells were transfected with Effectene transfection reagent (Qiagen), following the manufacturer’s instructions. For CPEB-KD induction, cells were treated or not with 1 mM IPTG (isopropyl-β-D-thio-galactoside) (Sigma) and analyzed to assess gene-silencing 3 days after induction.

### Inducible CPEBs knockdown cell lines

shRNA human CPEB1 was directed against 5’–GGTACTGAGCATGCTCCATAA - 3’ and cloned in the pLKO-puro-IPTG-3xLacO lentiviral vectors (Sigma Aldrich), shRNA human CPEB2 TRCN0000150178 (number assigned by the by the RNAi Consortium, TRCN) cloned in the pLKO-puro-IPTG-3xLacO, shRNA human CPEB3 TRCN0000240700 cloned in the pLKO-puro-IPTG-3xLacO, shRNA human CPEB4 was directed against 5’-GCTGCAGCATGGAGAGATAGA- 3’ and cloned in the pLKO-puro-IPTG-3xLacO. Virus production was performed as previously described [6]. shRNA mouse CPEB1 was directed against 5’- CCATCTTGAATGACCTATTTG-3’, shRNA human CPEB2 TRCN0000339452 cloned in the pLKO-puro-IPTG-3xLacO, shRNA human CPEB3 TRCN0000240700 cloned in the pLKO-puro-IPTG-3xLacO, shRNA human CPEB4 was directed against 5’-GCTGCAGCATGGAGAGATAGA-3’.

HEK-293 and H5V cells were infected with the different IPTG-inducible shRNA-producing viruses. Cells were induced (shRNA+) or not (shRNA-) with 1 mM IPTG every day for 3 days for subsequent analysis. For protein, RNA, flow cytometry analysis and determination of DNA content, shRNA-expressing cells were DTB-synchronized between days 1 and 3 of treatment with 1 mM IPTG, and samples were collected on day 3 of IPTG addition. For DNA content analysis, cells were DTB-synchronized (between days 1 and 3 of treatment with 1 mM IPTG), and samples were fixed on day 3 of IPTG addition.

### Transient CPEBs knockdown cell lines

shRNA human CPEB1 TRCN0000149470 cloned in the pLKO.1, shRNA human CPEB2 TRCN0000146533 cloned in the pLKO.1, shRNA human CPEB3 TRCN0000240697 cloned in the pLKO.1, shRNA human CPEB4 TRCN0000156565 cloned in the pLKO.1. HEK-293 cells were transfected using Lipofectamine 3000 (Life Technologies) according to the manufacturer’s protocols. After 48 hours, cells were selected in 3μg/ml puromycin (Life Technologies) for 48 hours.

### Synchronization of HEK-293 cells

Synchronization at the G1/S border was done by double thymidine (Sigma) treatment (17 h with 2 mM thymidine, 8 h release, and 16 h with 2 mM thymidine). Cells were collected at 0, 2.5, 5, and 9 h after release. A small fraction of cells was fixed with ethanol, stained with propidium iodide, and analyzed by flow cytometry to confirm cell cycle phase. Small fractions of cells were used to investigate proteins and mRNA levels.

### Synchronization of H5V cells

Synchronization at the G1/S border was done by double thymidine (Sigma) treatment (13 h with 2 mM thymidine, 9.5 h release, and 15.30 h with 2 mM thymidine). Cells were collected at 0, 2.5, 5, and 9 h after release. A small fraction of cells was fixed with ethanol, stained with propidium iodide, and analyzed by flow cytometry to confirm cell cycle phase. Small fractions of cells were used to investigate proteins and mRNA levels.

### Cell extracts and Western blot analysis

Cells were lysed in ice-cold Triton buffer (20mM HEPES pH 7.0, 150mM NaCl, 1% Triton X-100, 10% glycerol, 1mM EDTA, 1mM phenylmethylsulphonyl fluoride, 1 X protease inhibitors (Sigma). Lysates were centrifuged at 16,000*g*, and supernatants were resolved by 4–20% Criterion precast polyacrylamide gel (Bio-Rad). 30 ug of lysate was loaded onto each lane.

### qPCR

Total RNA was isolated using the RNAspin Mini Kit (GE Healthcare 25-0500-72) and additionally treated with TURBO DNA-free Kit (Ambion inc). Reverse transcription-PCR was performed using the RevertAid^TM^ First Strand cDNA Synthesis Kit (K1622 Fermentas), following the manufacturer’s instructions. mRNA levels for CPEB1, CPEB2, CPEB3, CPEB4, GFP, and RFP were measured by qRT-PCR and normalized to the internal control, α-galactosidase. The relative quantitation of each mRNA was performed using the comparative Ct method. PCR was carried out in a LightCycler 480 (Roche) using SYBRGreen I Master (Roche) and the primers indicated in the ‘oligonucleotides’ section above. Gene expression was monitored by amplifying constitutive exons. Each experiment was performed in triplicate.

### Poly(A) tail assay

Poly(A) tail assay was performed as previously described [6], with some modifications. Total RNA from HEK-293 cells was extracted using the RNAspin Mini Kit, additionally treated with the TURBO DNA-free Kit, and precipitated with Lithium Chloride at final concentration of 2.5 M. Next, 6 μg of total RNA was incubated for 30 min at 37°C in the presence of 0.2 μl of oligo (dT), 20 μM, and 0.2 U of RNase H (M0297S, New England Biolabs) or H2O. RNA was extracted by Phenol Chloroform purification. Next, 4 μg of RNA was ligated to 0.4 μg of SP2 anchor primer in a 10 μl reaction using T4 RNA ligase (New England Biolabs M0204L), following the manufacturer’s instructions. The whole 10 μl RNA ligation product was used in a 50 μl reverse transcription performed with the RevertAid^TM^ First Strand cDNA Synthesis Kit using 0.4 μg ASP2T antisense primer. 1 μl of cDNA was used to perform 50 μl PCR reactions using BioTaq Polymerase (Bio21040, Bioline). PCR products were resolved in 2% agarose gel.

### Flow cytometry

GFP and RFP levels were analyzed by FACS (Gallios flow cytometer, Beckman Coulter). Cells were sorted by FACS Aria III cell sorter (BD Biosciences). DNA content was stained with propidium iodide as indicated [18], and the samples were analyzed by FACS (Gallios flow cytometer, BD). The proliferation assay was assayed by measuring EdU incorporation using the Click-iT Plus EdU Alexa Fluor 647 Flow Cytometry Assay kit (Life Technologies), following the manufacturer’s instructions. The DNA content was measured with propidium iodide and analyzed by FACS (Gallios flow cytometer, BD). Flow cytometry data were analyzed with FlowJo software (Tree Star, Inc). For data analysis the mean of the GFP and RFP signal of RFP^+^/GFP^+^ cells was plotted at each time point.

### Microscopy

For live imaging analysis, cells were seeded in a four-compartment CellView^TM^ cell culture dish (Greiner Bio One) in a humidified 5% CO2 atmosphere at 37C on a temperature controlled spinning disk confocal microscope (Andor). Images were captured every 10 minute. Acquisition was optimized for minimum laser power such that mitotic timings were not perturbed by imaging.

Histone-H2B transfected cells were plated in a six-well glass bottom dish (MatTek) and analyzed by automated inverted microscope (TIRF, ScanR Olympus). Images were processed using ImageJ software. Mitotic index analysis was conducted using GraphPad Prism.

## Results

### 1. CPEB1, 2 and 4, but not CPEB3, are required for cell cycle progression

To define a suitable cellular model to perform a comparative analysis of all four CPEBs during cell-cycle progression, we first measured the mRNA expression levels of CPEB1-4 in a number of cell lines. We found that transformed cells of distinct origin, tissue and species, expressed different ratios of CPEB mRNAs. HeLa cells expressed mainly CPEB1 and 4, with little or no CPEB2 and CPEB3 [6]. H5V cells, expressed mainly CPEB1, with intermediate levels of CPEB2-4. HEK-293 expressed equivalent levels of all four CPEBs (Fig A in S1 File). Since HEK-293 showed expression of all four CPEB mRNAs, these were chosen as the cellular model to determine the requirement of each CPEB during the cell cycle.

**Fig 1:**
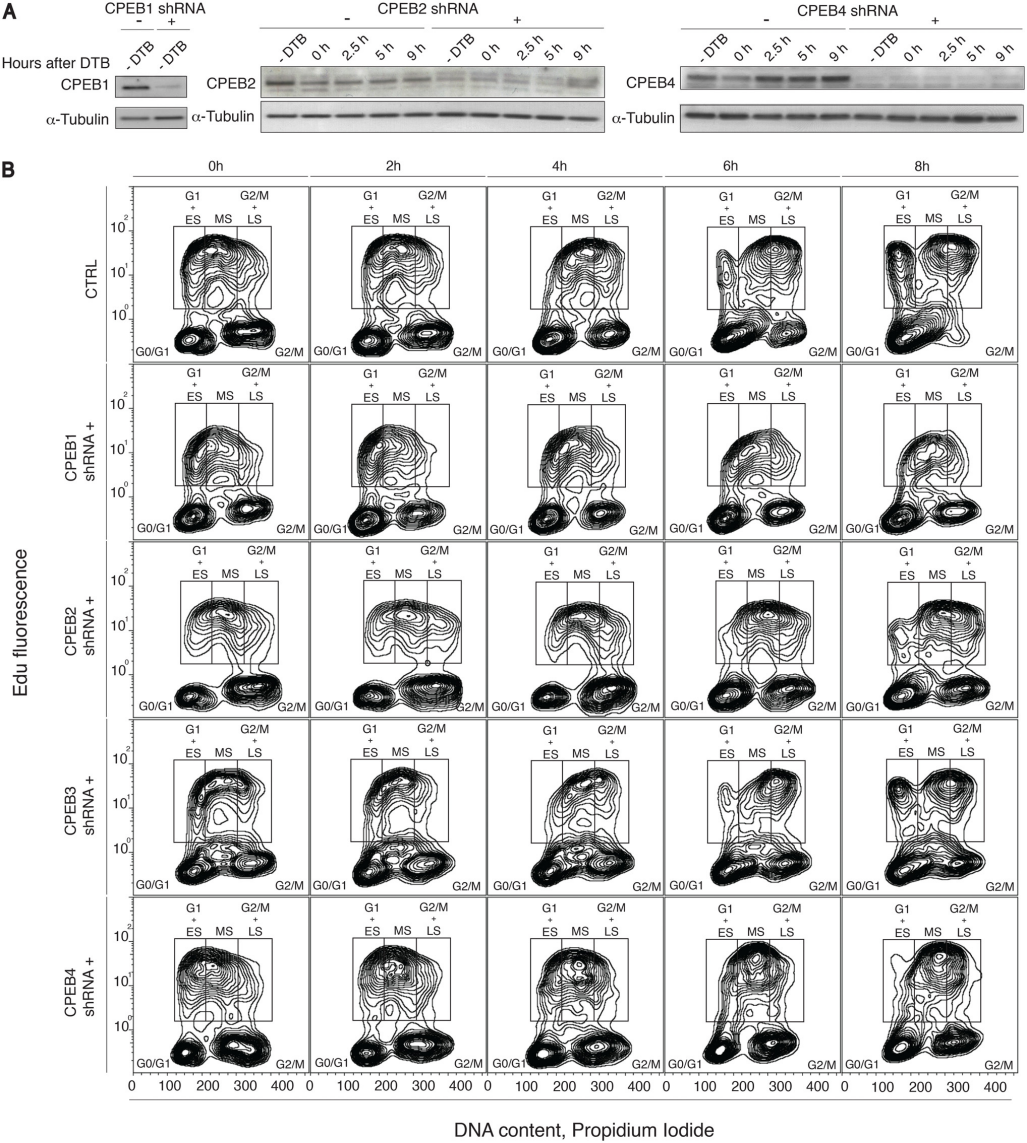
CPEB1, CPEB2 and CPEB4 are required for cell cycle progression. (A) HEK-293 cells stably expressing an IPTG-inducible system for CPEB1,2 and 4 knock-down were induced or not with IPTG. Two days after induction cells were synchronized through double thymidine blockade (DTB). Samples were harvested at the indicated time points and protein lysates were analyzed on SDS–PAGE followed by immunoblotting for the indicated proteins. α-tubulin was used as a loading control. (B) HEK-293 cells stably expressing an IPTG-inducible system for each CPEB knock-down were induced or not with IPTG. Three days after induction, cells were marked with EdU for two hours and then released. Samples were harvested at the indicated time points, stained for DNA content with PI and analyzed by FACS. Mean EdU^+^ cell-population: 23100 for control, 10800 for CPEB1-KD cells, 16400 for CPEB2-KD cells, 20900 for CPEB3-KD cells, 18900 for CPEB4-KD cells). Sh, short-hairpin; CTRL, control; IPTG, Isopropyl β-D-1-thiogalactopyranoside; DTB, double-thymidine block; Edu, 5-ethynyl-2'-deoxyuridine; PI, propidium iodide; ES, early S-phase; MS, middle S-phase; LS, late S-phase; FACS, fluorescence-activated cell sorter.

Four HEK-293 CPEB inducible knock down cell lines were stably generated using shRNAs against each CPEB (see methods for details). Each cell line expresses an Isopropyl β-D-1-thiogalactopyranoside (IPTG)-inducible CPEB-specific shRNA to knock down each of the four CPEBs individually. Using RT-qPCR, we measured relative mRNA levels for each CPEB after three days of IPTG-induction (Fig B in S1 File). For CPEB1, CPEB2 and CPEB4, we confirmed the knock down efficiency by immunoblotting (Fig 1A). In the case of CPEB3, the knock down could only be detected by qPCR, as CPEB3 antibodies (commercial or generated in the laboratory) did not render a specific signal.

To assess the expression of the CPEB proteins across the cell cycle, we synchronized the cells by double thymidine blockade (DTB = early S phase), and analyzed DNA content at four time points after release, at the G1/S boundary, S-phase, G2/M phase, and at the G1 phase of the control cells (Fig C in S1 File). Interestingly, as compared to control, CPEB1-KD cells were unresponsive to thymidine treatment, and showed the same distribution of cells in the cell cycle phases at all time points (Fig C in S1 File, compare Control panel to CPEB1 shRNA+ panel), thereby indicating a major defect in cell cycle progression and/or checkpoints. CPEB3-KD cells were comparable to control cells; CPEB2-KD and CPEB4-KD cells showed an increased distribution of cells in G2/M phases (Fig C in S1 File, compare 9h time points, grey bars, from Control panel, 16.8%, to CPEB2, 26.4%, and CPEB4 shRNA+, 23.8%). These cell cycle impairments had only a modest impact on cell viability and only for CPEB2 KD did we observe a significant increase in cell death (Fig D in S1 File).

To further analyze the impact of CPEBs depletion on cell cycle progression, we performed 5-ethynyl-2´-deoxyuridine (EdU) pulse-chase experiments. Control or CPEB-KD cells were pulsed with Edu for two hours, cultured in Edu-free media for 8 hours and fixed at the indicated time points (Fig 1B). After propidium iodide (PI) staining, we analyzed them by fluorescence-activated cell sorting (FACS). In control cells, the Edu-pulsed cells at the early S phase were chased into G2/M-phases for 8 hours, whereas the cells pulsed in late S reached G1 by this time. By contrast, CPEB1-KD, Edu-pulsed cells in early S phase displayed almost no progression through S-phase and into G2/M phases, even after 8 hours of chase, confirming a strong proliferation defect. CPEB2-KD and CPEB4-KD cells showed a delayed progression through cell cycle, with a significant number of Edu-pulsed cells still in mid S-phase, and no Edu^+^ cells in G1, after 8 hours of chase. A large proportion of both CPEB2-KD and CPEB4-KD cells were blocked at G2 and M phases. CPEB3-KD cells did not show any significant defects in proliferation or cell cycle, with a phase distribution during the chase indistinguishable from that of the control cells. Altogether, these results indicate that depletion of CPEB1 produces a severe block of cell proliferation (clearly observed also by microscopy, S2 Video and Fig G in S1 File), whereas depletion of CPEB2 or CPEB4 causes a delayed entry or progression through M-phase. CPEB3 depletion did not cause any defect in cell cycle progression. Thus, in HEK-293 cells, CPEB1, 2 and 4 but not CPEB3 appeared critical for normal cell cycle progression.

### 2. Phase-specific requirements for CPEB1, 2, and 4 in mitosis

**Fig 2:**
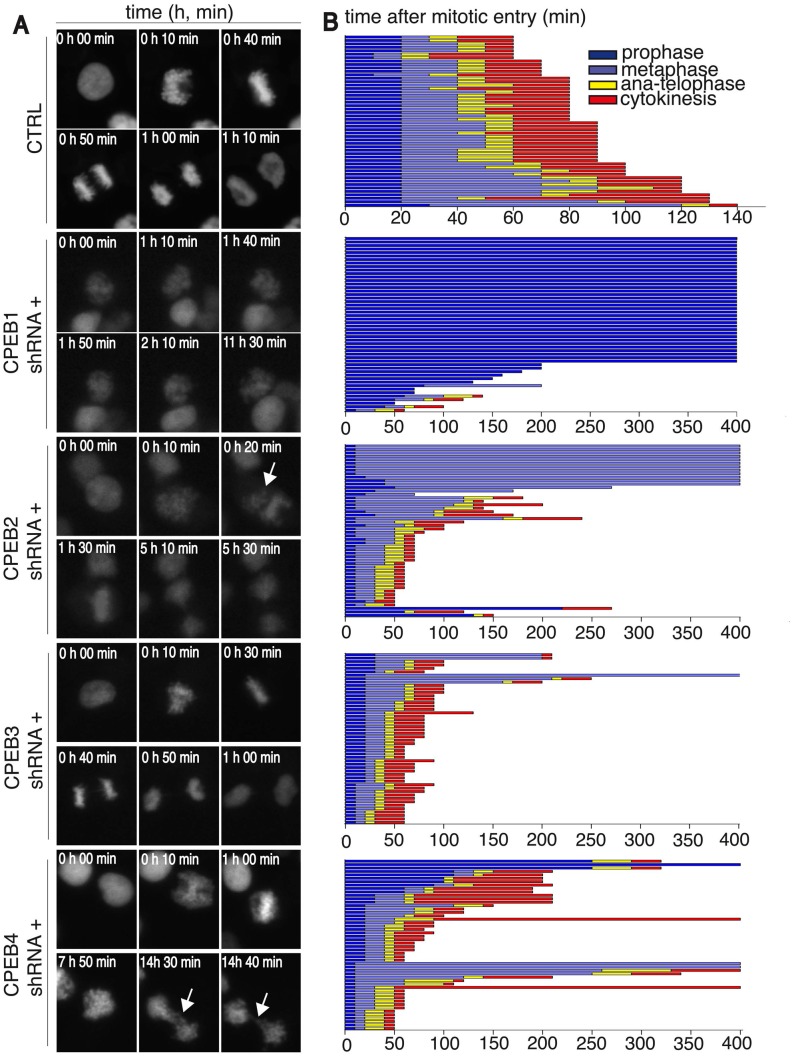
CPEB1 is required for prophase entry; CPEB2 for metaphase-to-anaphase transition and CPEB4 for cytokinesis. (A) HEK-293 cells stably expressing an IPTG-inducible system for each CPEB knock-down were induced or not with IPTG. Two days after induction, cells were transfected with a plasmid encoding fluorescent histon H2B. After one additional day cells were recorded by live imaging experiments. Images were acquired every 10 minutes and analyzed for mitotic progression. Representative images are shown for individual cells in interphase or during specific phases of mitosis that are readily identified by chromosome condensation state and organisation. (B) Mitotic-stage analysis of 50 cells from S1–S5 Videos as in Fig 2A. On Y axis, each lane represents one cell. On X axis, time is represented as minutes. Colors represent the indicated mitotic phases. Mitotic entry was determined by analyzing the first signs of DNA condensation cross-reinforced with cell-rounding. Mitotic exit was scored based on chromosome segregation at anaphase and DNA decondensation. Sh, short-hairpin; CTRL, control.

To further define the cell-cycle defects caused by individual CPEB depletion, we followed the mitotic chromosome dynamics, through the transient overexpression of red fluorescent protein-histone H2B (RFP-H2B) fusion protein in each of the four CPEBs-KD cell lines (Fig 2A, S1–S5 Videos). We first calculated the percentage of cells in mitosis (mitotic index, Fig E in S1 File). We then used the H2B signal to determine the number of cells in each mitotic-phase (see methods for details) [i.e. for Fig 2A control cells: decondensed (time 0, interkinesis), condensed (time 10’, prophase), organized metaphase plate (time 40’, metaphase), segregating chromosomes (times 50’ and 1h; anaphase and telophase) and de-condensing chromatin (1h 10’; cytokinesis)]. In some cases (Fig 2A, white arrows), RFP-histone H2B revealed abnormal chromatin organization (quantified in Fig E in S1 File). Finally, for 50 mitotic cells in each condition, we quantified the time spent in each mitotic phase (Fig 2B).

CPEB3-KD cells showed no major defects (Fig 2A and S4 Video, Fig 2B for quantification). Cells expressing CPEB1 shRNA exhibited the most severe cell cycle phenotype, with a mitotic index significantly lower than in control cells (3.6%, Fig E in S1 File). Interestingly, almost all the mitotic CPEB1-depleted cells were restricted to prophase (Fig 2A and S2 Video, Fig 2B for quantification). In addition, CPEB1-KD cells failed to reattach, forming clumps partially or totally detached from the cell culture plates, thereby suggesting that CPEB1 is also required for cell/matrix attachment. This observation may be related to the fact that CPEB1 regulates the expression of Fibronectin [14]. In contrast, CPEB2-KD cells showed remarkable defects in mitotic progression and were restricted mainly to metaphase. Their metaphase plates were misshapen, with chromosomes not properly condensed and aligned (Fig 2A panel CPEB2 shRNA+), and the cells spent more time in metaphase before progressing to anaphase (Fig 2A and S3 Video, Fig 2B for quantification—please mind X-axis scale is different from control cells). In addition, we observed a small number of cells with abnormal chromatin arrangement and defects in cytokinesis (Fig 2B). CPEB4-KD cells had major defects during telophase/cytokinesis (Fig 2B panel CPEB4). Despite some defects in metaphase plate formation, only a few cells spent more time in this mitotic phase. Instead, they progressed through anaphase and telophase, during which we observed the most frequent and noticeable defects in cytokinesis (Fig 2A, white arrows and S5 Video, Fig 2B for quantification). More than 6% of CPEB4-KD cells showed abnormal chromosome segregation (Fig E in S1 File). In order to rule out off-target effects and confirm the specificity of the inducible-KD system, we transiently transfected an additional set of shRNAs for CPEB1-4 and observed the same phenotypes (Figs F and G in S1 File). Overall, these data demonstrate that CPEB1, CPEB2, and CPEB4 have distinct roles in normal mitosis and their deletion causes disruption of the normal process of mitosis. Thus, CPEB1 is required for prophase entry, CPEB2 for metaphase, and CPEB4 for cytokinesis, suggesting that these three proteins have sequential and non-redundant function during mitosis.

### 3. CPEB1, CPEB2, and CPEB4 are required for CPE-mediated translational regulation at G2-M phases

Given the non-redundant roles CPEB proteins play in mediating normal progression of mitosis and that CPEBs can act both as translational repressors or activators, we next sought to understand if the translational regulation of mRNAs with CPE-containing 3’UTRs was differentially affected by each CPEB depletion at any specific phase of the cell cycle.

For this purpose, in each CPEB-KD cell line, we stably co-transfected two constructs: one encodes a destabilized green fluorescent protein, fused to a CPEs-containing 3’UTR (GFP-3’UTR+CPE, Fig H in S1 File) and the other encodes for a destabilized red fluorescent protein, followed by a control 3’UTR (RFP-3’UTR CTRL, Fig H in S1 File) [19]. Double positive cells were sorted to ensure cells containing both constructs were isolated. GFP and RFP mRNA abundance did not change significantly during the cell cycle nor as result of the knockdown inductions, indicating that changes in GFP and RFP expression are due to translational regulation (Fig H in S1 File).

**Fig 3:**
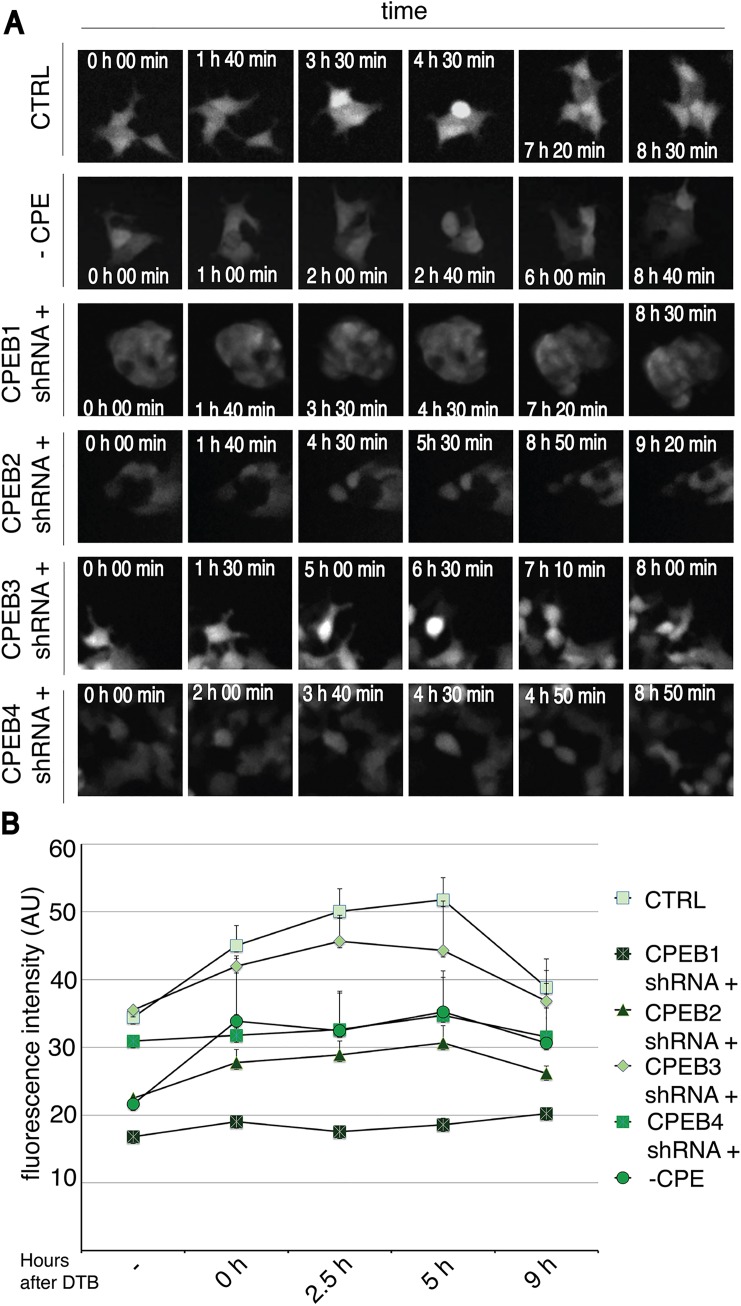
CPEB1, CPEB2 and CPEB4 are required for proper GFP translation at G2 and M phases. (A) HEK-293 cells stably expressing an IPTG-inducible system for each CPEB knock-down and carrying a GFP-3'UTR +/-CPE together with a RFP-3'UTR were induced or not with IPTG. Two days after induction, cells were recorded by live imaging experiments and analyzed for GFP expression (see Fig I in S1 File, for RFP expression). Representative images from S6–S11 Videos are shown. (B) HEK-293 cells stably expressing an IPTG-inducible system for each CPEB knock-down and cells carrying a GFP-3'UTR with mutated CPE (CPE-) were induced or not with IPTG. Two days after induction cells were synchronized by double thymidine blockade (DTB). Samples were collected at the indicated phases of the cell cycle and GFP expression was measured by FACS. Results are shown as the mean value from six experiments, error bars indicate s.d; au, arbitrary units; Sh, short-hairpin; CTRL, control.

We then analyzed the relative protein expression of GFP and RFP during the cell cycle using two complementary strategies. Single cells were analyzed through live imaging experiments and their morphology was also assessed (Fig 3A and Fig I in S1 File show, for each KD condition, GFP and RFP levels were quantified during the cell cycle by fluorescence-activated cell sorting (FACS) of cells synchronized by DTB (Fig 3B and Fig I in S1 File). Through FACS measurements we privileged statistical power at cost of resolution (due to the relative efficiency of cell synchronization and relative stochasticity of GFP expression) but we could gain high statistical power.

Live imaging of control cells demonstrated over 30% of cells (Fig J in S1 File) with a transient burst of GFP protein expression starting just before cell division and ceasing once cells had divided (Fig 3A, CTRL, 4h 30 min and S6 Video). Instead, RFP levels remained constant during the cell cycle (Fig I in S1 File and S6 Video). Indicating that translational activation of CPE-regulated mRNAs takes place at late G2 and M phases of the cell cycle. FACS analysis of control cells confirmed this increase in GFP expression, which peaked 5 hours after release from the DTB (corresponding to the G2 and M phases, Fig 3B), RFP levels remained constant during cell cycle (Fig I in S1 File). Given that GFP and RFP RNAs do not change during the cell cycle (Fig H in S1 File), these results suggest that the CPE-containing 3’UTR was controlling GFP translation at G2/M.

In order to explore whether this phenotype was mediated by CPEs, we induced point mutations in their sequences. CPEs inactivation prevented the burst of GFP expression, as shown by live imaging (Fig 3A, S11 Video) and FACS (Fig 3B), indicating a CPE-dependent translational activation concomitant with the G2/M transition. To further define the CPEB(s) responsible for this regulation, we induced CPEBs knock down and analyzed GFP and RFP levels. Live imaging (Fig 3A, S7 Video) and FACS analysis (Fig 3B) of CPEB1-KD cells showed no transient activation of GFP, suggesting that CPEB1 is required for translational activation of GFP. CPEB2- and CPEB4-KD cells were defective in increasing GFP translation before division (Fig 3A, S8 and S10 Videos). In fact only 2% of CPEB2-KD cells and 4% of CPEB4-KD cells showed this specific phenotype (Fig J in S1 File). FACS analysis showed that, when these two proteins are individually depleted, the levels of GFP across the cell cycle were similar to the control without CPEs (Fig 3B). These results suggest that CPEB2 and CPEB4 are required for CPE-mediated translational activation of GFP at G2/M phases. Conversely, GFP level in cell expressing CPEB3-sh was indistinguishable from that of controls, both when analyzed by live imaging (Fig 3A, S9 Video) and FACS (Fig 3B). These observations suggest that CPEB3 is not implicated in CPE-mediated translational regulation during cell cycle progression.

### 4. CPEB1, CPEB2, and CPEB4 coordinate cytoplasmic polyadenylation dynamics during the cell cycle

We next addressed whether the cell-cycle-specific translational regulation of the GFP reporter mRNA was mediated by changes in its polyadenylation.

**Fig 4:**
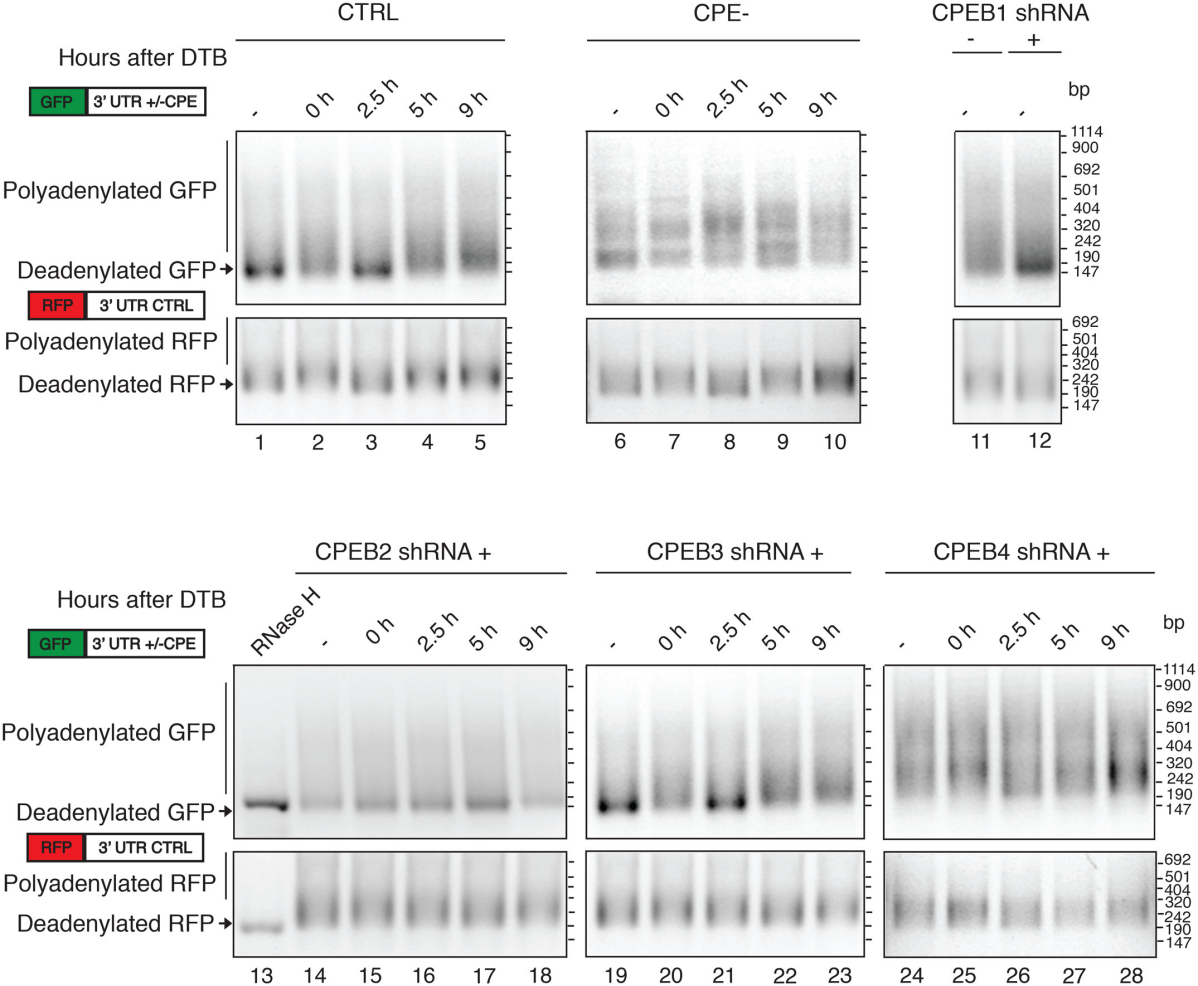
CPEB1, CPEB2 and CPEB4 are required to coordinate proper polyadenylation dynamics during the cell cycle. HEK-293 cells stably expressing an IPTG-inducible system for each CPEB knock-down and cells carrying a GFP-3'UTR with mutated CPE (CPE-) were induced or not with IPTG. Two days after induction cells were synchronized through double thymidine blockade (DTB). Samples were collected at the indicated time points after DTB release. Total RNA was extracted in order to analyze GFP and RFP poly(A) tail length. Digestion with RNAse H (lane 13) indicates the deadenylated GFP and RFP mRNAs. The positions of the deadenylated and polyadenylated RNAs are indicated. Schematic representation of the transfected constructs is shown. GFP open reading frame in green, RFP open reading frame in red. 3’UTRs, 3’-untranslated region; CPE, cytoplasmic polyadenylation element; CTRL, control.

We purified mRNAs at each phase of the cell cycle and analyzed the GFP and RFP poly(A) tail length (Fig 4). While the RFP poly(A) tail maintained a constant length during the cell cycle, the CPEs-containing GFP reporter mRNA (CTRL) showed cell cycle-dependent changes in poly(A) tail length. In the asynchronous control cells, short GFP poly(A) tails were observed then progressing to intermediate lengths at the G1/S border (0h), short lengths at the S phase (2.5h), and progressively longer tails during the G2/M and G1 phases (5 and 9 h). The polyadenylation pattern of the CPE-mutated GFP mRNA was different. It did not change during the cell cycle, indicating that these changes were dependent on the presence of CPE, and poly(A) tails were generally long, maintaining the long poly(A) tails acquired co-transcriptionally, and therefore indicating that CPEs are required for deadenylation of the mature transcript as well as for later readenylation at specific cell cycle phases. When the CPEB proteins were knocked down, such polyadenylation dynamics were affected in different manners. In CPEB1-KD cells, the major population of GFP mRNAs was deadenylated (Fig 4). CPEB2-KD cells retained short GFP poly(A) tails throughout all the cell cycle phases (Fig 4, lanes 13 to 17). In contrast, CPEB4-KD cells showed GFP poly(A) tails that were consistently longer than those of the controls (Fig 4A, lanes 24 to 28). CPEB3-KD cells showed a pattern of GFP polyadenylation similar to that of control cells (Fig 4, lanes 19 to 23).

Our results indicate that phase-specific regulation of cytoplasmic polyadenylation during cell cycle is achieved through the coordination of CPEB1, CPEB2, and CPEB4 functions. Proper polyadenylation dynamics are established only when they function in concert. However, although CPEBs are required both for cell cycle specific translational control and polyadenylation of the CPE containing reporter mRNA, the differential effect of CPEB2-KD and CPEB4-KD suggests that the lengthening of the poly(A) tail is necessary but not sufficient to contribute to mRNA translation. In fact, coexistence of specific poly(A) tail elongation dynamics (i.e. mRNA polyadenylation at specific moments of the cell cycle), rather than the absolute poly(A) tail length itself, appears to be required to ensure the effective flow of information from mRNAs to proteins. Moreover, CPEB4-KD cells show polyadenylation defects, hyperadenylation, at 2.5 h, suggesting CPEB4 may already play a role in inactivating mRNAs during S-phase (or S to G2 phase transition). However, no major phenotype was observed at this phase of the cell cycle indicating that this CPEB4-mediated translational translational repression is required for latter, M-phase, cell cycle progression events. Interestingly, CPE inactivation led to variable GFP levels (Fig 3B) and polyadenylation (Fig 4, lanes 6 to 10), thereby suggesting that CPEB-mediated regulation of cytoplasmic polyadenylation and mRNA translation is required to prevent noisy gene expression along the cell cycle.

## Discussion

The CPEB-family of RNA binding proteins are expressed, with defined but overlapping patterns, in most differentiated tissues. However, their relative contribution to cell division is not yet known. Thus, CPEB1 and CPEB4 are essential regulators of maternal mRNAs during meiotic reactivation in transcriptionally silent oocytes, but it is not known if CPEB2 or CPEB3 are also implicated. CPEB1 supports either cell proliferation or senescence in non-embryonic cells, but it is not known what defines this differential effect or how its activity is coordinated with CPEB2-4. Given that all CPEBs bind the same *cis*-acting element [5] and regulate the same or overlapping mRNAs [2], defining the function(s) of individual CPEBs requires understanding how the members of the family interact functionally in a cellular context where all four are expressed.

To define whether all or specific CPEBs are required for mitotic cell cycle progression and to determine the extent to which they exhibit a functional interplay (by compensation, sequential functions, synergies, etc.), we performed a systematic depletion of all four CPEBs in a transformed cell line, HEK-293, that expresses similar levels of all four proteins (at the mRNA level). We then measured the requirement of each CPEB for cell cycle progression, phase-specific translational activation of a CPE-regulated mRNA, and phase-specific cytoplasmic polyadenylation in dividing cells.

Our results show that CPEB3 is the only member of the family not required for mitotic progression and that it is not required for polyadenylation or translational activation of CPE-regulated mRNAs. The other three members, CPEB1, CPEB2, and CPEB4, are all required for cell cycle progression, phase-specific regulation of poly(A) tail length and translational activation of CPE-regulated transcripts. Although for CPE-regulated mRNAs, we do not observe a correlation between deadenylation and destabilization during cell cycle [6], but rather with translational efficiency, this behavior may be specific of CPEB-targets as for other transcripts and in asynchronous cells poly(A) tail length correlates with mRNA half-life, but not with translational efficiency [20, 21].

CPEB1, CPEB2 and CPEB4 appear to have sequential non-redundant functions and thus cannot compensate for each other. Therefore, when CPEB1, 2, and 4 act in concert, the poly(A) tails of CPE-regulated transcripts become progressively longer while approaching the G2 and M phases. Once the cells divide, the reporter mRNA-poly(A) tail progressively shortens to start another cycle of cell cycle-specific cytoplasmic polyadenylation. This G2/M-specific phase of cytoplasmic polyadenylation triggers protein translation, as demonstrated by the GFP burst prior to cell division. Depletion of CPEB1 blocks this phase specific polyadenylation (Fig 4) and the subsequent translational activation (Fig 3B), causing arrest of cell division at the G2/M transition. CPEB1-depleted cells also detach from the substrate, which causes some events of arrest at other phases, even if minor compared with the prophase-entry phenotype (Fig 2B). This suggests that CPEB1, in addition to cell cycle, regulates mRNAs promoting cell-to-matrix attachment (as for fibronectin [14]). CPEB2 depletion also impedes the polyadenylation and translational activation of CPE-regulated transcripts at the G2/M phases (Fig 4 and Fig 3B, respectively); however, in this case, the cells arrest at metaphase (Fig 2B). This is the first time a mitotic function for CPEB2 is described. Finally, CPEB4 depletion causes accumulation of polyadenylated CPE-regulated mRNAs during the whole cell cycle (Fig 4) with a sustained translation in all phases and no burst (Fig 3B). These cells display cytokinesis defects.

In HEK-293 cells CPEBs mRNAs were expressed to similar levels (Fig A in S1 File). However the relative abundance of each CPEB may play a crucial role in the coordination of cell cycle progression. H5V mouse endothelial cells have high CPEB1 mRNA levels and relatively lower levels of the other CPEBs (Fig A in S1 File). In these cells we found that CPEB1 knock down have a minor influence on cell cycle progression and translation of the GFP/RFP reporter, whether CPEB2- and CPEB4-KD have a similar effect to the one observed in HEK-293 cells (Figs K, L and M in S1 File). This is most likely due to differential levels of depletion, as a factor of the higher levels of CPEB1, when compared with CPEB2 and 4, as CPEB1 depletion has been shown to cause mitotic defects in other cell lines beyond HEK-293 [6]. As for HEK-293 cells, in H5V cells CPEB3 does not seem to be involved in the regulation of cell cycle progression (Figs K, L and M in S1 File).

## Conclusions

Overall we found that in HEK-293 cells all three CPEBs (1, 2 and 4) functions are sequential and phase-specific, with CPEB1 acting first, probably promoting the cytoplasmic polyadenylation and expression of factors required for metaphase entry, followed by CPEB2, which sustains this polyadenylation and translational activation during metaphase. Thus CPEB2 is required for metaphase completion and exit. Finally, CPEB4 is required for the deadenylation of these mRNAs and completion of mitosis.

## Supporting Information

S1 File **Fig A. HEK-293 expressed equivalent levels of all four CPEBs.** (A) Total mRNA was purified from H5V cells and mRNAs retrotranscribed using oligo(dT). CPEB1-4 were amplified using specific oligos. Relative mRNA levels are represented. Error bars indicate s.d.
(B) Total mRNA was purified from HEK-293 cells and mRNAs retrotranscribed using oligo(dT). CPEB1-4 were amplified using specific oligos. Relative mRNA levels are represented. Error bars indicate s.d. **Fig B. CPEB1-4 knock-down quantification by RT-qPCR.** HEK-293 cells stably expressing an IPTG-inducible system for each CPEB knock-down were induced or not with IPTG. Total mRNA was purified and mRNAs retrotranscribed using oligo(dT). CPEB1-4 were amplified using specific oligos. Relative mRNA levels are represented. Error bars indicate s.d. **Fig C. Control and CPEB1-4 knock-down cells were synchronized by double thymidine blockade.** HEK-293 cells stably expressing an IPTG-inducible system for each CPEB knock-down were induced or not with IPTG. Two days after induction cells were synchronized through double thymidine blockade (DTB). Samples were harvested at the indicated time points after release and fixed in 70% EtOH. DNA content was measured by PI staining. Percentages of cells in G1, S, and G2/M (a mixed population of G2 and mitosis) are included. Results are shown as the average of five experiments. **Fig D. Cell death analysis of control and CPEB1-4 knock-down cells by staining with propidium iodide.** HEK-293 cells stably expressing an IPTG-inducible system for each CPEB knock-down were induced or not with IPTG. Three days after induction, cell-viability was assessed by propidium iodide staining. The fold change in dead cell number, as compared to control cells, is plotted. Results are shown as the mean value of three experiments, error bars indicate s.d. **Fig E. Mitotic index of asynchronous control and CPEB1-4 knock-down cells.** HEK-293 cells stably expressing an IPTG-inducible system for each CPEB knock-down were induced or not with IPTG. Two days after induction, cells were transfected with a plasmid encoding fluorescent histone H2B. After one additional day cells were recorded by live imaging experiments. Images were acquired every 10 minutes and analyzed for mitotic progression. Mitotic index was calculated on the acquired images. The percentages of cell with normal mitosis (white) and abnormal chromosome segregation (grey) were determined (n = 1000). Results are shown as the mean value from three experiments. **Fig F. Transient CPEBs knock down in HEK-293 cells**. HEK-293 cells were transiently transfected with CPEB1, 2, 3 and 4 shRNA constructs, as indicated in “Materials and Methods”. Two days after transfection the indicated samples were harvested and protein or RNA lysates were analyzed on SDS–PAGE followed by immunoblotting for the indicated proteins (A) or by qPCR (B). **Fig G. CPEB1 is required for prophase entry; CPEB2 for metaphase-to-anaphase transition and CPEB4 for cytokinesis.** (A) HEK-293 cells transiently expressing an shRNA for each CPEB were induced or not with IPTG. After one day, cells were transfected with a plasmid encoding fluorescent histon H2B. After one additional day cells were recorded by live imaging experiments. Images were acquired every 10 minutes and analyzed for mitotic progression. Representative images are shown for individual cells in interphase or during specific phases of mitosis that are readily identified by chromosome condensation state and organization. (B) Mitotic-stage analysis of 50 cells. On Y axis, each lane represents one cell. On X axis, time is represented as minutes. Colors represent the indicated mitotic phases. Mitotic entry was determined by analyzing the first signs of DNA condensation cross-reinforced with cell-rounding. Mitotic exit was scored based on chromosome segregation at anaphase and DNA decondensation. Sh, short-hairpin; CTRL, control. **Fig H. RT-qPCR of GFP and RFP transcripts from synchronous control and CPEB1-4 knock-down cells.** (A) Schematic representation of the transfected constructs is shown. GFP open reading frame in green, RFP open reading frame in red. 3’UTRs stands for “3’-untranslated region”, “CPE” stands for “cytoplasmic polyadenylation element”, CTRL stands for “control”. (B) HEK-293 cells stably expressing an IPTG-inducible system for each CPEB knock-down and cells carrying a GFP-3'UTR with mutated CPE (CPE-) were induced or not with IPTG. Two days after induction cells were synchronized through double thymidine blockade (DTB). Samples were harvested at the indicated time points and total RNA was extracted. Quantitative PCR for GFP and RFP from reverse transcribed mRNAs (RT). Results are shown as the mean value from three experiments, error bars indicate s.d. **Fig I. CPEBs do not regulate RFP translation during the cell cycle.** (A) HEK-293 cells stably expressing an IPTG-inducible system for each CPEB knock-down and carrying a GFP-3'UTR +/-CPE together with a RFP-3'UTR were induced or not with IPTG. Two days after induction, cells were recorded by live imaging experiments and analyzed for RFP expression. Representative images from S6–S11 Videos are shown. (B) HEK-293 cells stably expressing an IPTG-inducible system for each CPEB knock-down and cells carrying a GFP-3'UTR with mutated CPE were induced or not with IPTG. Two days after induction cells were synchronized by double thymidine blockade (DTB). Samples were collected at the indicated phases of the cell cycle and RFP expression was measured by FACS. Results are shown as the mean value from six experiments, error bars indicate s.d; au, arbitrary units. **Fig J. Quantification of GFP burst observed in control and CPEB1-4 knock-down cells by live imaging.** HEK-293 cells stably expressing an IPTG-inducible system for each CPEB knock-down and cells carrying a GFP-3'UTR with mutated CPE (CPE-) together with a RFP-3'UTR were induced or not with IPTG. Two days after induction, cells were recorded by live imaging experiments and analyzed for GFP and RFP expression (control). Number of GFP burst prior to cell division was counted in each condition. The percentage of cells showing such GFP burst is plotted (n = 100). **Fig K. Inducible CPEBs knock down in H5V cells.** H5V cells stably expressing an IPTG-inducible system for CPEB1, 2, 3 and 4 knock-down were induced or not with IPTG. Four days after induction the indicated samples were harvested and protein or RNA lysates were analyzed on SDS–PAGE followed by immunoblotting for the indicated proteins (A) or by qPCR (B). **Fig L. Control and CPEB1-4 knock-down H5V cells were synchronized by double thymidine blockade.** H5V cells stably expressing an IPTG-inducible system for each CPEB knock-down were induced or not with IPTG. Two days after induction cells were synchronized through double thymidine blockade (DTB). Samples were harvested at the indicated time points after release and fixed in 70% EtOH. DNA content was measured by PI staining. Percentages of cells in G1, S, and G2/M (a mixed population of G2 and mitosis) are included. Results are shown as the average of three experiments. **Fig M. CPEBs regulate GFP but not RFP translation during the cell cycle of H5V cells.** H5V cells stably expressing an IPTG-inducible system for each CPEB knock-down and cells carrying a GFP-3'UTR with mutated CPE were induced or not with IPTG. Two days after induction cells were synchronized by double thymidine blockade (DTB). Samples were collected at the indicated phases of the cell cycle and the GFP (A) or RFP (B) expression was measured by FACS. Results are shown as the mean value from three experiments, error bars indicate s.d; au, arbitrary units.
(DOCX)

S1 Video Video related to Fig 2A: H2B-GFP fluorescent time lapse microscopy of CTRL cells. (AVI)

S2 Video Video related to Fig 2A: H2B-GFP fluorescent time lapse microscopy of cells expressing CPEB1 shRNA+. (AVI)

S3 Video Video related to Fig 2A: H2B-GFP fluorescent time lapse microscopy of cells expressing CPEB2 shRNA+. (AVI)

S4 Video Video related to Fig 2A: H2B-GFP fluorescent time lapse microscopy of cells expressing CPEB3 shRNA+. (AVI)

S5 Video Video related to Fig 2A: H2B-GFP fluorescent time lapse microscopy of cells expressing CPEB4 shRNA+. (AVI)

S6 Video Video related to Fig 3A and Fig I in S1 File: GFP/RFP fluorescent time lapse microscopy of CTRL cells. (AVI)

S7 Video Video related to Fig 3A and Fig I in S1 File: GFP/RFP fluorescent time lapse microscopy of cells expressing CPEB1 shRNA+. (AVI)

S8 Video Video related to Fig 3A and Fig I in S1 File: GFP/RFP fluorescent time lapse microscopy of cells expressing CPEB2 shRNA+. (AVI)

S9 Video Video related to Fig 3A and Fig I in S1 File: GFP/RFP fluorescent time lapse microscopy of cells expressing CPEB3 shRNA+. (AVI)

S10 Video Video related to Fig 3A and Fig I in S1 File: GFP/RFP fluorescent time lapse microscopy of cells expressing CPEB4 shRNA+. (AVI)

S11 Video Video related to Fig 3A and Fig I in S1 File: GFP/RFP fluorescent time lapse microscopy of cells expressing GPF ORF with CPE– 3’ UTR (CPE -). (AVI)
