# Supplementary material for: Global Analysis of CPEBs Reveals Sequential and Non-Redundant Functions in Mitotic Cell Cycle
Source: PLoS One. 2015 Sep 23;10(9):e0138794. doi: 10.1371/journal.pone.0138794 (PMC4580432; doi:10.1371/journal.pone.0138794)
Supplement: S1 File — Fig A. HEK-293 expressed equivalent levels of all four CPEBs. (A) Total mRNA was purified from H5V cells and mRNAs retrotranscribed using oligo(dT). CPEB1-4 were amplified using specific oligos. Relative mRNA levels are represented. Error bars indicate s.d. (B) Total mRNA was purified from HEK-293 cells and mRNAs retrotranscribed using oligo(dT). CPEB1-4 were amplified using specific oligos. Relative mRNA levels are represented. Error bars indicate s.d. Fig B. CPEB1-4 knock-down quantification by RT-qPCR. HEK-293 cells stably expressing an IPTG-inducible system for each CPEB knock-down were induced or not with IPTG. Total mRNA was purified and mRNAs retrotranscribed using oligo(dT). CPEB1-4 were amplified using specific oligos. Relative mRNA levels are represented. Error bars indicate s.d. Fig C. Control and CPEB1-4 knock-down cells were synchronized by double thymidine blockade. HEK-293 cells stably expressing an IPTG-inducible system for each CPEB knock-down were induced or not with IPTG. Two days after induction cells were synchronized through double thymidine blockade (DTB). Samples were harvested at the indicated time points after release and fixed in 70% EtOH. DNA content was measured by PI staining. Percentages of cells in G1, S, and G2/M (a mixed population of G2 and mitosis) are included. Results are shown as the average of five experiments. Fig D. Cell death analysis of control and CPEB1-4 knock-down cells by staining with propidium iodide. HEK-293 cells stably expressing an IPTG-inducible system for each CPEB knock-down were induced or not with IPTG. Three days after induction, cell-viability was assessed by propidium iodide staining. The fold change in dead cell number, as compared to control cells, is plotted. Results are shown as the mean value of three experiments, error bars indicate s.d. Fig E. Mitotic index of asynchronous control and CPEB1-4 knock-down cells. HEK-293 cells stably expressing an IPTG-inducible system for each CPEB knock-down were in [file pone.0138794.s001.docx]

**SUPPORTING INFORMATION**

**Global analysis of CPEBs reveals sequential and non-redundant functions in mitotic cell cycle**

Valeria Giangarrà^1,#a^, Ana Igea^1^ , Chiara Lara Castellazzi^1^, Felice-Alessio Bava^1,#b*^ and Raul Mendez^1,2*^

^1^ Institute for Research in Biomedicine (IRB Barcelona), Barcelona, Spain

^2^ Institució Catalana de Recerca i Estudis Avançats (ICREA), Barcelona, Spain

^*^Corresponding authors

E-mail: [raul.mendez@irbbarcelona.org](mailto:raul.mendez@irbbarcelona.org) (RM)

E-mail: [bava@stanford.edu](mailto:bava@stanford.edu) (F-AB)

^#a^Current address: Stanford University School of Medicine, Pediatric Cardiac Surgery Division, Stanford, California, United States of America

^#b^Current address: Stanford University, Baxter Laboratory in Stem Cell Biology, Stanford, California, United States of America

**Figure A**

**Figure B**

**
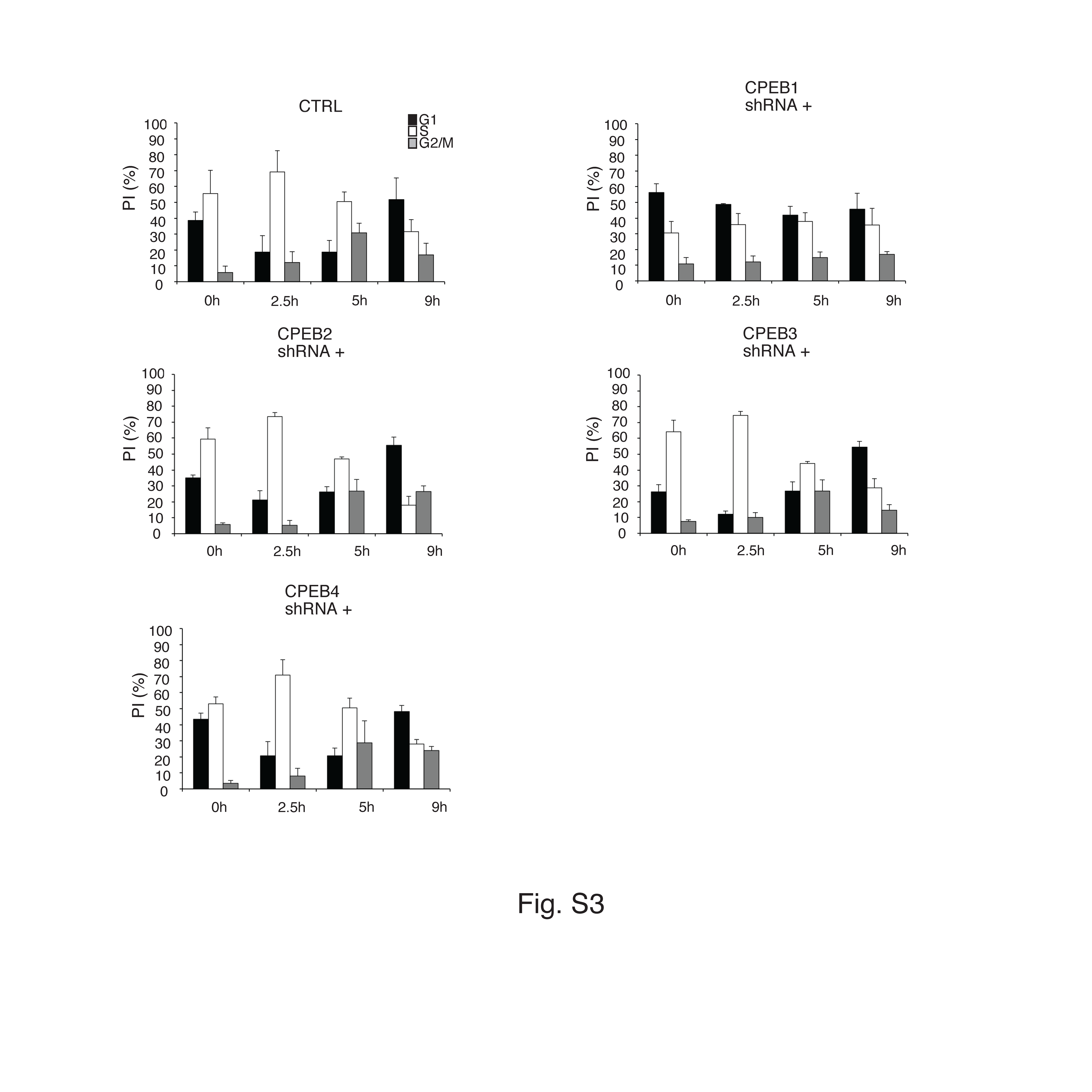
**

**Figure C.**

**
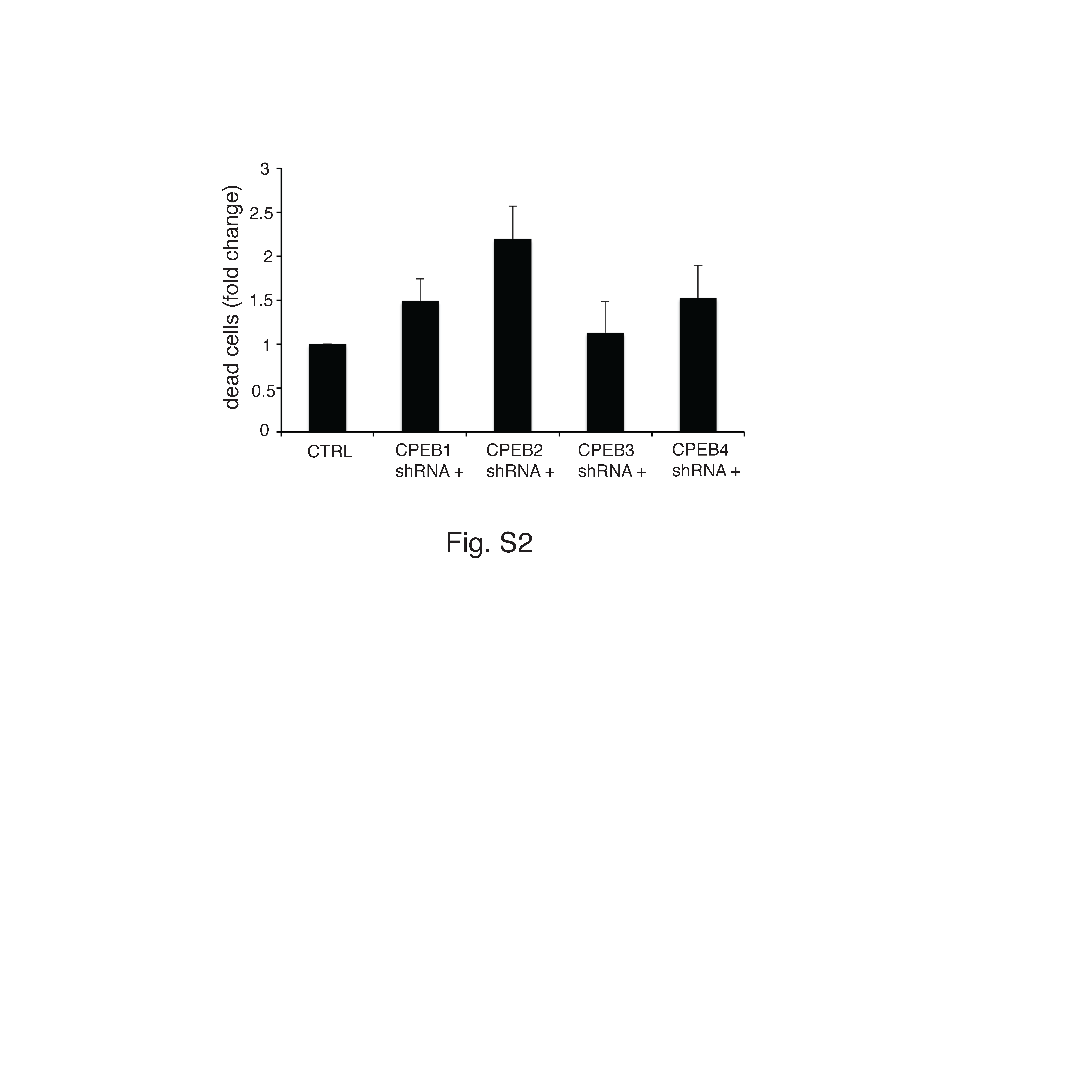
**

**Figure D**

**Figure E**

**Figure F**

**
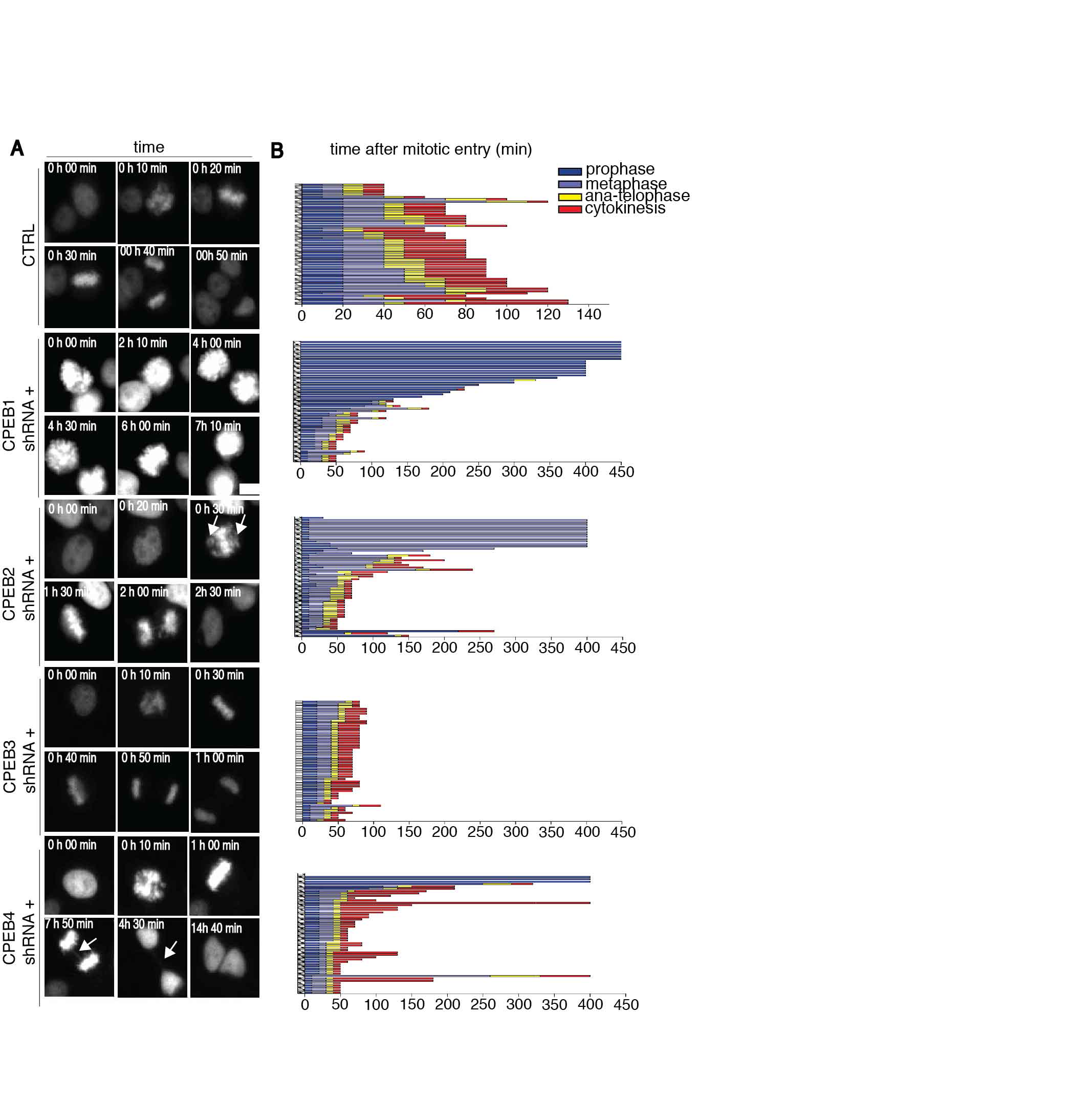
Figure G**

**Figure H.**

**Figure I**


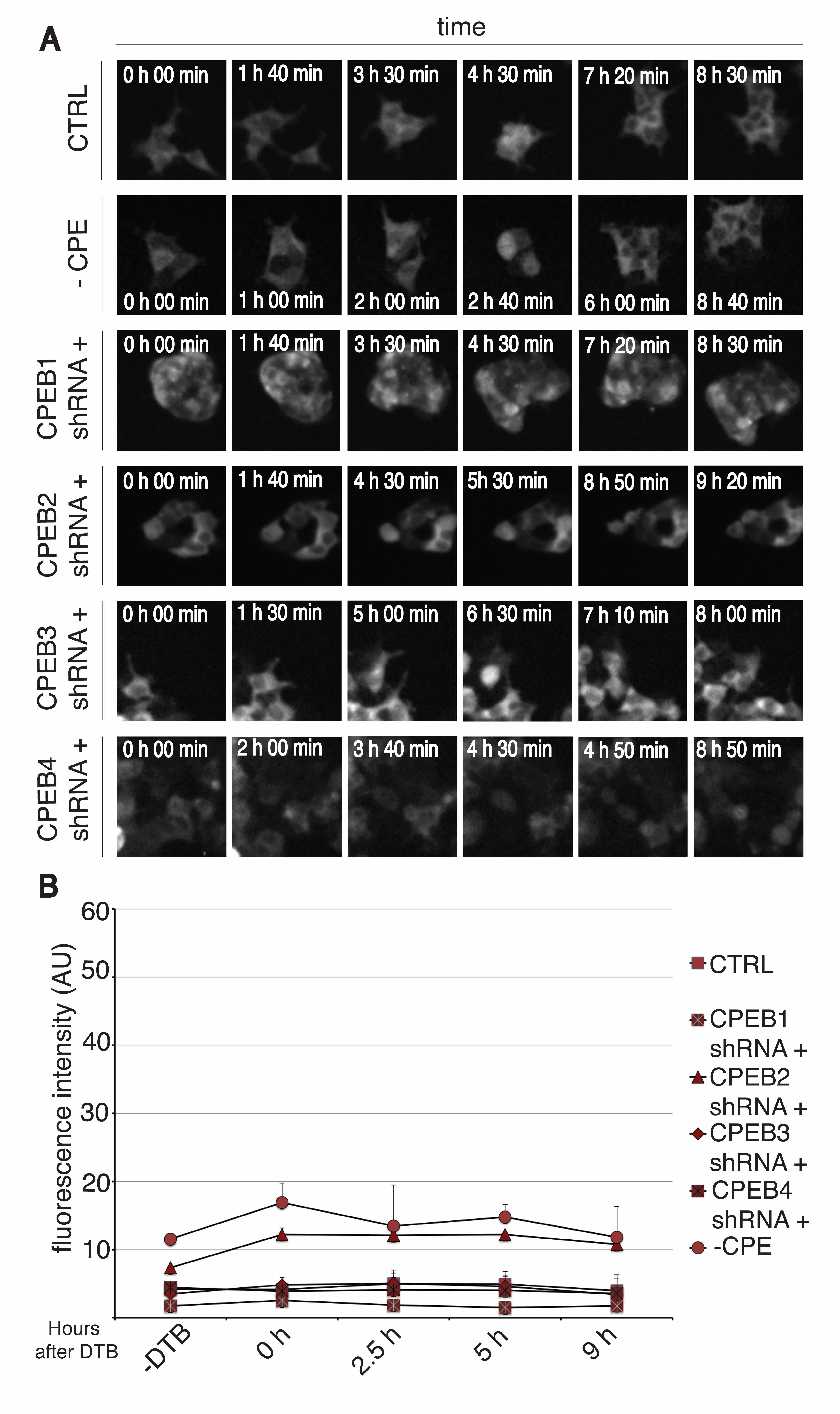


**
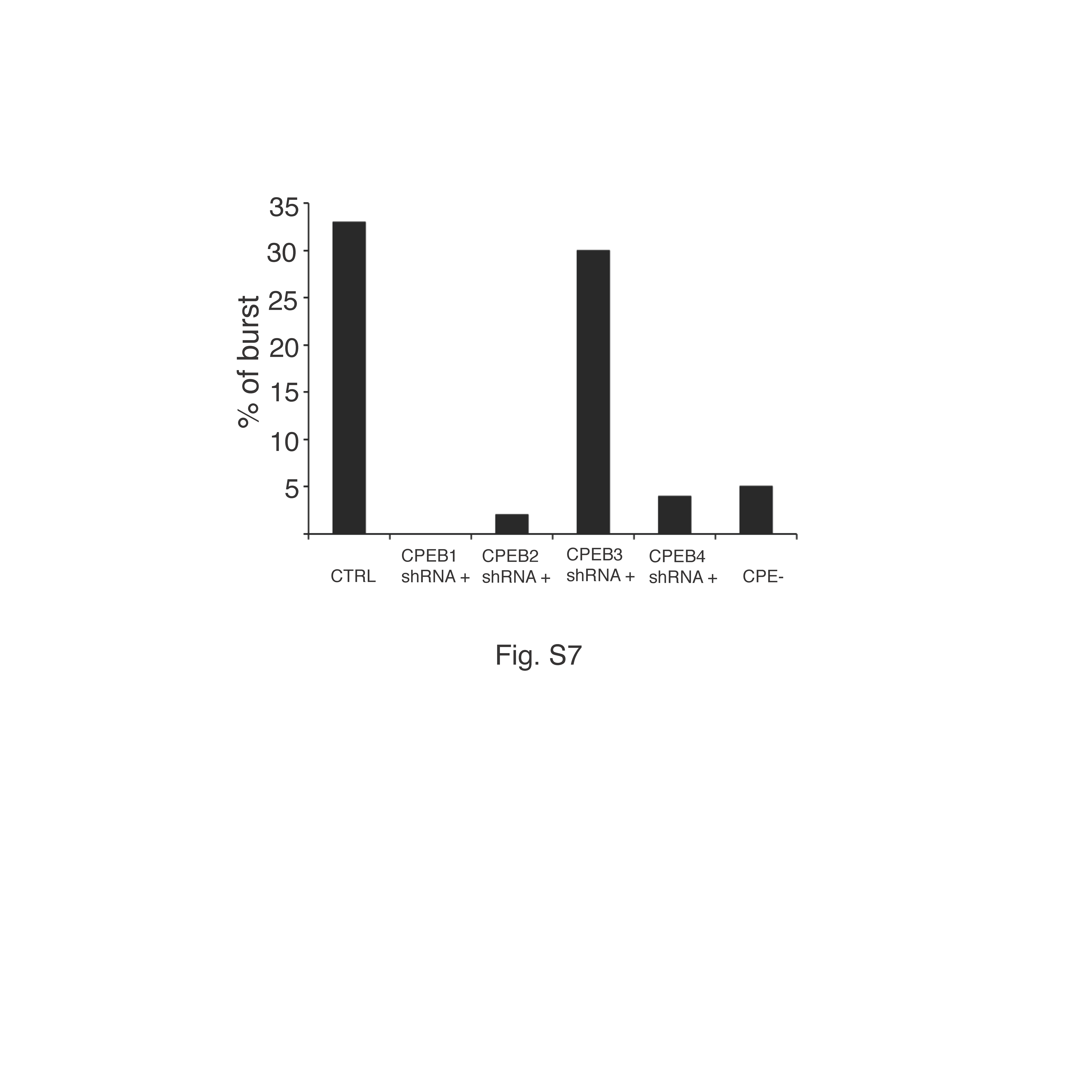
Figure J.**

**
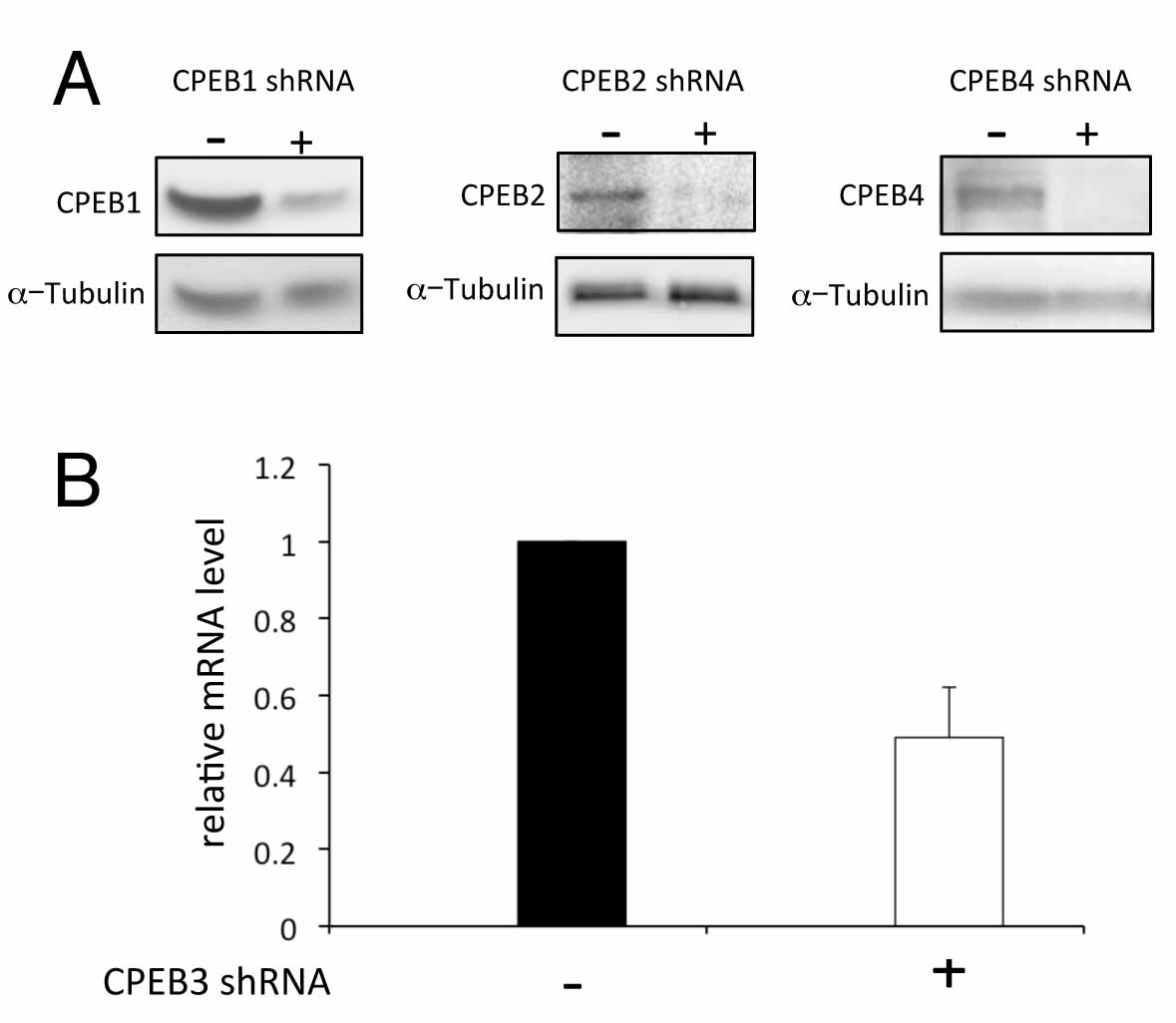
Figure K**

**Figure L**

**Figure M**
